# Invasive *Klebsiella pneumoniae* Infections, California, USA

**DOI:** 10.3201/eid1609.100386

**Published:** 2010-09

**Authors:** Robert McCabe, Larry Lambert, Brad Frazee

**Affiliations:** Author affiliations: Alameda County Medical Center, Oakland, California, USA (R. McCabe, L. Lambert, B. Frazee);; St. Rose Hospital, Hayward, California, USA (R. McCabe)

**Keywords:** Hypermucoviscous, Klebsiella pneumoniae, string test, invasive infection, bacteria, California, abscess, letter

**To the Editor:** A distinctive form of tissue-invasive community-associated *Klebsiella pneumoniae* infection, typified by primary liver abscess and bacteremia, has been well known in Asia for 2 decades (*1**–**4*). Association of these infections with a hypermucoviscous phenotype was discovered in 2004 (*5*). Certain genetic and virulence features were elucidated in that and subsequent reports (*6*).

The phenotype, easily detected at the bench by the string test (*5*), has been associated with a chromosomal gene, the mucoviscosity-associated gene A (*magA*), and a plasmid gene, the regulator of the mucoid phenotype A gene (*rmpA*). Usually serotypes K1 and K2 can be demonstrated. Hypermucoviscous isolates demonstrate increased virulence in mice, are serum insensitive, and resist phagocytosis (*5*).

Reports of such infections from Europe and North America are rare. Recently 2 of us (L.L and B.F.) reported 4 cases in persons seeking care at the Alameda County Medical Center in Oakland, California, USA (*7*). We report 9 more cases, 7 from Alameda County Medical Center and 2 from St. Rose Hospital in Alameda County. The 13 cases are described in aggregate.

One case occurred in 2006, 3 in 2007, 7 in 2008, and 2 in January 2009. Median patient age was 52 years (range 37–70 years), and 9 were men. Ten patients were born in Asia (Philippines, Vietnam, South Korea, Cambodia, and Yemen), but all had emigrated years earlier. Two patients were born in the United States (1 Filipino and 1 African American); the birth site of 1 Filipino was unknown. Five patients had no underlying illness. Seven had diabetes mellitus, 1 had α-thalassemia, 2 had uncontrolled cancer, and 1 had preexisting multiple organ failure. Three patients had gallstones.

Case-patients exhibited diverse clinical features. The site of infection was easily detected by dramatically abnormal results of computed tomographic scan or magnetic resonance imaging (Figure). Seven patients had liver abscesses. One of these patients also had cholecystitis and choledocholithiasis. One other patient with healthcare-associated bacteremia most likely had multiple small liver abscesses that were superinfected cancer metastases.

**Figure:**
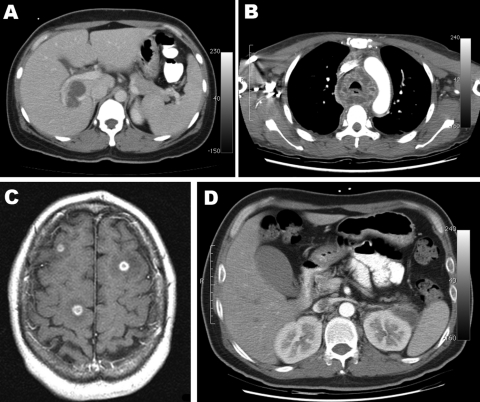
A) Computed tomography (CT) scan of the abdomen showing a liver abscess adjacent to the portal vein. B) CT scan of the chest at the level of the aortic arch showing mediastinum abscesses surrounding the trachea. C) Brain magnetic resonance imaging (T1 weighted, spin echo, with contrast) showing multiple intracerebral abscesses (smooth ring-enhancing lesions with surrounding vasogenic edema). D) CT scan of the abdomen of patient from panel C, showing a left perinephric abscess and thrombus.

Two patients had neck abscesses, 1 complicated by extensive descending mediastinitis (Figure). One patient had kidney abscesses complicated by septic and bland pulmonary emboli and numerous brain abscesses detected by magnetic resonance imaging (Figure).

Healthcare-associated bacteremia occurred in 3 patients. One patient had sustained bacteremia without a clear source on hospital day 115. Two patients with uncontrolled cancer also had healthcare-associated bacteremia.

Venous thrombotic complications occurred in 6 patients, most diagnosed at admission. Two patients had bland pulmonary emboli, and 1 patient with uncontrolled cancer had thrombosis of an upper extremity vein and both femoral veins. Two other patients had septic pulmonary emboli suggested by computed tomographic scan. One patient with α-thalassemia had kidney abscesses and renal vein thrombosis, followed by femoral deep vein thrombosis and pulmonary embolus. A patient with a neck abscess had a thrombosed neck vein at surgery.

Four patients died, and 1 was lost to follow-up, for a death rate of at least 31%. None died directly of sepsis.

All isolates were resistant in vitro to ampicillin but susceptible to all other antimicrobial drugs tested. Genotyping was performed on isolates from 4 patients. One isolate contained the *rmpA* gene; 3 contained *rmpA* and *magA* genes. Three of these isolates also underwent capsule serotyping; 2 were type K1 and 1 was K2.

We found 4 additional patients infected with *K*. *pneumoniae* in 2009 who did not have invasive infections. Briefly, a 21-year-old pregnant recent emigrant from Yemen and a 35-year-old Hispanic pregnant woman each had a urinary tract infection; a 78-year-old Vietnamese man had nosocomial aspiration pneumonia in which *K*. *pneumoniae* was considered a pathogen; and a 34-year-old African American woman who was receiving mechanical ventilation had sputum transiently colonized with *K*. *pneumoniae.*

This case series confirms that the clinical syndrome of *K. pneumoniae* bacteremia and primary liver abscess has emerged in Alameda County. Other sites of infection included kidney, brain, lung, pleural space, neck, and mediastinum, as reported in Asia (*1**–**4*). Although *K. pneumoniae* infections are predominantly a community-associated phenomenon, nosocomial infections as we observed have been reported (*8*). Death reflected underlying disease rather than *K*. *pneumoniae* infection in this study. We present evidence that hypermucoviscous *K*. *pneumoniae* also can behave as a colonizer or low-virulence pathogen, as manifested in our patients with urinary tract infection, sputum colonization, and aspiration pneumonia.

Our *K*. *pneumoniae* isolates appear similar to those from Asia (*5*) with respect to in vitro susceptibility, capsule serotypes, and *magA* and *rmpA* genes. Most of our patients were Asian but of widely dispersed origin and without recent travel to Asia. The number of thrombotic complications in this series is striking. Such complications appear not to have been noted in the literature, and this finding requires further investigation. Our data show the emergence of hypermucoviscous *K. pneumoniae* in northern California and suggest that it might be unrecognized elsewhere in North America.
